# Which Is the Ideal Treatment for Benign Diffuse and Multinodular Non-Toxic Goiters?

**DOI:** 10.3389/fendo.2016.00048

**Published:** 2016-05-23

**Authors:** Meyer Knobel

**Affiliations:** ^1^Thyroid Unit, Division of Endocrinology and Metabolism, University of São Paulo Medical School, Hospital das Clínicas, São Paulo, Brazil

**Keywords:** benign goiter, non-toxic, diffuse, multinodular, treatment

## Abstract

Patients with large benign goiters often present local compressive symptoms that require surgical treatment, including dysphagia, neck tightness, and airway obstruction. In contrast, patients with such goiters who remain asymptomatic may be observed after exclusion of malignancy. The use of levothyroxine (LT4) to reduce the volume of the goiter is still a controversial treatment for large goiters, and the optimal surgical procedure for multinodular goiter is still debatable. Radioiodine is a safe and effective treatment option when used alone or in combination with recombinant human TSH. This review discusses current therapeutic options to treat diffuse and multinodular non-toxic benign goiters.

## Introduction

Once the diagnosis of non-toxic diffuse goiter (NDG) or non-toxic nodular goiter (NNG) is established, the following management goals should be considered:

- Correct the underlying thyroid dysfunction, if present;
- Verify if the goiter is growing or causing obstructive symptoms;
- Exclude malignancy if one or more nodules are suspicious;
- Determine whether the goiter requires therapy, and if so, weight the benefits and risks of medical and surgical interventions and reach a decision with the patient about what type of treatment should be administered.

The expression of the progressive and nodular increase of the thyroid in non-toxic benign goiters is the result of a combination of genetic and environmental factors, of which iodine deficiency is the most important (1). The treatment of benign non-toxic goiter is a challenge for clinicians and endocrinologists. Similarly to how patients with single thyroid nodules are assessed, all individuals with benign non-toxic goiter must undergo careful evaluation with serum TSH measurement and thyroid ultrasonography to guide the selection of the nodule (or nodules) to be biopsied (2). After exclusion of malignancy, treatment should be considered for individuals with compression of local structures, cosmetic concerns, and/or thyroid hyperfunction (2). The choice of treatment in patients with benign non-toxic goiter can be challenging, considering that the size of the goiter and the occurrence of symptoms often follow a non-linear association (Table 1).

**Table 1:** **Treatment choices reported by ATA, ETA, and LATS members based on an index case of a patient with a simple non-toxic nodular goiter, without suspicion of malignancy**.

**Therapy**	**Non-toxic nodular goiter**		
	**ATA (*n*** **= 140)**	**ETA (*n*** **= 120)**	**LATS (*n*** **= 148)**
None	36	28	39
Levothyroxine	56	52	21
Radioiodine^a^	1	6	7
Surgery	6	10	28

*The numbers correspond to the percentage of organization members who responded to the questionnaire*.

*ATA, American Thyroid Association; ETA, European Thyroid Association; LATS, Latin American Thyroid Association*.

*Adapted from Ref. (3)*.

*^a^Includes both isolated radioiodine use and radioiodine associated with rhTSH*.

### Non-Toxic Diffuse Goiter

There is no ideal therapy for NDGs (4), but for patients requiring treatment, clinical management is the most frequent choice. It is unclear whether or not early treatment of NDGs can inhibit the development of nodular goiter.

The development of NDGs occurs from a combination of genetic and environmental factors. Prolonged TSH stimulation, which frequently occurs in association with iodine deficiency, has an important role in thyroid enlargement. In these circumstances, iodine supplementation would be an adequate approach (400 μg of iodine for 8–12 months). A significant reduction in goiter size has been observed in patients with NDG supplemented with iodine (5, 6). In fact, supplementation with iodine 400 μg/day has been demonstrated to be as effective as suppressive therapy with LT4 150 μg/day (5). However, except in a few European countries, iodine is no longer used for the treatment of benign non-toxic goiter.

In contrast, a beneficial effect of LT4 has been shown in NDG (7). A volume reduction of 50% or more was detected in 31% (11/35) of the patients after 6 months (7). If suppressive treatment is considered, then administration of thyroid hormone in enough doses to inhibit or reduce TSH secretion may be used. However, it should be considered that the volume of the goiter returns to pretreatment size after LT4 withdrawal. As may be expected, many thyroid experts are unenthusiastic about treating NDG, although others advocate the use of LT4 favoring the notion that early treatment may prevent later development of nodular goiter. As with any issue in clinical medicine, the benefits of such therapy must be weighed against the potential risks of TSH suppression.

Regarding radioiodine therapy in NDG, two earlier small uncontrolled studies are available (8, 9). In the first study, 11 patients with symptoms of pressure and cosmetic complaints were selected for radioiodine ablation after refusing surgical treatment. The mean thyroid volume reduction achieved with a single dose of radioiodine within the first year was 62%, and 2 of the 11 patients (18%) developed hypothyroidism (8). In another study, 10 patients were treated with radioiodine and were followed up for 18 months with measurements of the thyroid volume by ultrasonography. The volume declined by 50% within 12–18 months. During follow-up, one patient (10%) developed persistent hypothyroidism and another (who presented positive antithyroperoxidase levels) developed transient hypothyroidism (9).

### Non-Toxic Nodular Goiter

The ideal treatment for NNG is controversial (Table 1) (3), in part due to the variation in the natural history of these goiters. Some patients present an enlargement in goiter size with time along with the development of nodules, symptoms of compression, and cosmetic reasons (10). In contrast, the enlargement of the goiter may become stable or reduce spontaneously with time in around 20% of the women and 5% of the men (11).

Current alternatives for NNG treatment include

- Clinical observation for asymptomatic patients;
- Thyroid hormone suppressive therapy;
- Radioiodine therapy alone or preceded by recombinant human TSH (rhTSH); and
- Surgery.

Among these options, treatment is chosen individually for each patient in view of the risks, benefits, and availability of the various techniques, experience of the treating physician, and patient’s personal preference (Table 2).

**Table 2:** **Therapeutic options for individuals with non-toxic multinodular goiter**.

**Treatment**	**Advantages**	**Disadvantages**	**Comments**
Levothyroxine	Outpatient use Low cost Prevent formation of other nodules?	Low efficacy Main effect on perinodular volume Continuous suppression of TSH, which leads to side events associated with subclinical hyperthyroidism, as well as unpredictable bone and cardiac side effects	Declining due to adverse effects and lack of efficacy
Surgery	Substantial reduction in goiter size Rapid relief of compression of vital cervical structures Allows pathological assessment	Not applicable to all individuals Post-surgical hemorrhage (1%) Damage to the recurrent laryngeal nerve (1–2%) Temporary (0.5%) or definitive (0.6%) hypoparathyroidism Goiter recurrence depending on the extent of resection Post-surgical tracheomalacia (rare) Increased morbidity in cases with large goiters, intrathoracic extension or reoperation	Standard treatment for large goiters or when rapid decompression of cervical structures is required Total thyroidectomy should be considered the preferred therapy to prevent goiter recurrence
Radioiodine	Thyroid volume reduction by half in 1 year Improves respiratory capacity in the long term Frequent outpatient use Can be successfully repeated Few side effects	Gradual reduction in goiter size The larger the goiter, the lower the effect Slight risk of short-term increase in goiter size 3% risk of thyroiditis 5% risk of development of Graves’ disease 15–20% risk of hypothyroidism at 12 months Small risk of radiation-induced ophthalmopathy Requires retreatment in some cases Risk of radiation-induced malignancy has not been established	In some European countries, radioiodine has become the standard therapy, replacing surgery May be considered in place of surgical intervention in patients who refuse or are unable to undergo surgery, or in those with large goiters (except in cases that require large radioiodine doses) Can be preceded by rhTSH with lower radioiodine dose

*Modified and adapted from Ref. (10)*.

## Clinical Observation

Clinical observation, including thyroid function monitoring and ultrasonographic assessment at regular intervals, is an alternative in cases of small goiters not causing compressive symptoms and associated with normal thyroid function. If clinical observation is chosen, the possibility of malignancy should be excluded by fine-needle aspiration biopsy (FNAB) that is guided by ultrasonography.

Asymptomatic euthyroid patients with benign non-toxic goiter and without cosmetic symptoms may be simply observed with clinical and laboratory evaluation and thyroid imaging tests. In most cases, patients with asymptomatic NNG may be evaluated yearly with careful thyroid palpation and measurement of serum TSH level. Ultrasonography may be performed when the palpation of the thyroid is uncertain, whereas nodules that appear to enlarge should be evaluated with FNAB. Evaluation with neck computed tomography or magnetic resonance imaging (MRI) may be required when the goiter is subesternal.

The American Thyroid Association recommends a standard follow-up interval of 6–18 months for patients with NNG, which may be gradually prolonged if no substantial changes are observed during the first 3–5 years (12).

## Suppressive Therapy with Levothyroxine

Although LT4 is broadly used in the United States, Europe, and Latin America, as shown in different surveys (3), the use of thyroid hormones to treat NNGs is controversial, and its efficacy is dependent on the degree of TSH suppression. Considering that NNG patients frequently have normal serum TSH levels, the enlargement of the thyroid in these patients is probably associated with the prolonged action of different growth factors (including TSH) on thyroid follicular cells with different synthetic and growth potentials. Advantages of this treatment modality include low cost, administration on an outpatient basis, and inhibition of the development of new nodules. In contrast, suppressive therapy has little effectiveness, as it requires permanent treatment and may have potential undesirable effects on bone (demineralization) (13) and heart (arrhythmias), especially in older individuals (14).

In a randomized placebo-controlled trial, a ≥25% goiter volume reduction was seen in 58% of the individuals with NNG and NDG during LT4 suppressive therapy over 9 months, with a return to the original volume after treatment withdrawal. Over the same period, patients randomized to the placebo group showed a 20% increase in thyroid volume (15).

In another randomized trial comparing suppressive therapy with radioiodine, patients in the radioiodine group showed a 35% reduction in goiter volume at 1 year and 44% at 2 years, whereas those who received LT4 presented 7 and 1% reduction at 1 and 2 years, respectively. A response to the therapy was found in 97% of the individuals who received radioiodine and 43% of those undergoing LT4 therapy (16). A recent meta-analysis showed lack of substantial benefits and a relative risk of only 1.9 (95% confidence interval, 0.95–3.81) of a decrease in nodular size with LT4 therapy (17). Thus, according to these randomized controlled trials, it seems that thyroid hormone suppression can interfere with the process of goitrogenesis (goiter growth and new nodule formation), with only some goiters responding and others growing despite LT4 treatment (15).

The occurrence of adverse events is more evident in older patients, a population that comprises most patients with NNG. Also, about 22% of the individuals with NNG may harbor areas with functional autonomy, which increase the concerns with LT4 therapy because of risks of bone loss and atrial fibrillation. Therefore, treatment with thyroid hormone should be avoided, especially in patients with prior serum TSH concentrations below the normal range.

Patients who start LT4 suppression must continue the therapy for a long time. The reduction in goiter size with LT4 seen in some individuals is probably due to decreased TSH secretion, especially in patients who live in areas with borderline or low iodine levels. Any decrease in NNG size that may occur with LT4 suppression is lost when the treatment is interrupted, and the nodules and goiter may grow again in size (15). In addition, keeping the serum TSH concentration in the lowest reference range or slightly below the normal range, rather than below 0.01 μU/mL, appears to be efficacious. Pending further studies, this strategy may relieve the adverse effects of LT4 therapy (18). Accordingly, an LT4 dose adjusted to obtain a non-suppressive level of serum TSH in the range of 0.5–0.8 μU/mL has been shown to significantly reduce the growth of nodules within multinodular goiters in 165 out of 356 female patients during a follow-up period of 9 years (19).

A potential benefit of thyroid hormone therapy is a reduction in the risk of thyroid oncogenesis (20). However, this hypothesis is still speculative, and more studies are still needed.

In general, since the volume of the thyroid reduces in only 30% of the patients with NNGs treated with LT4 suppression, recommendations for this type of treatment have decreased (10).

## Surgery

The best surgical procedure to treat patients with NNG is still controversial (21). A recent meta-analysis that included 1305 participants evaluated the impact of total/near-total thyroidectomy versus subtotal thyroidectomy in adults with non-toxic multinodular goiter. The key results of this study were (a) the recurrence of goiter was lower in patients who underwent thyroidectomy compared with those who underwent subtotal thyroidectomy, with rates of goiter recurrence of 84 in 1000 patients with subtotal thyroidectomy and 5 in 1000 patients with total thyroidectomy; (b) no clear benefits or harms were observed with subtotal or total thyroidectomy in patients undergoing surgical intervention due to recurrence in goiter size, complications such as permanent recurrent laryngeal nerve palsy, or occurrence of thyroid carcinoma; and (c) cancer detection was lower (6.1%) in patients undergoing subtotal thyroidectomy compared with 7.3% of those undergoing total thyroidectomy, but this difference was not significant (21).

In most patients with large obstructive goiters, cosmetic complaints, or retrosternal NNGs for whom surgery is recommended, total thyroidectomy is preferable over subtotal thyroidectomy (22, 23). In the long term, patients undergoing subtotal thyroidectomy have higher recurrence rates than those undergoing total thyroidectomy, and 2.5–42% are required to undergo a new intervention. In addition, 3.5% of the NNG patients undergoing subtotal thyroidectomy require another surgical intervention to remove remaining thyroid tissue because of incidental thyroid carcinoma (24). Rates of permanent complications, such as hypoparathyroidism and vocal palsy, are similar with both total and subtotal surgeries; however, total thyroidectomy is preferred due to an increased risk of these complications associated with a new intervention (25). In patients with unilateral NNG, some authors recommend unilateral thyroidectomy based on a low rate of recurrence (2%) and high rate of maintenance of euthyroidism (73%) (26).

Post-surgical recurrence rates are directly proportional to the volume of the remaining thyroid tissue. Hegedüs et al. (27) studied 202 consecutive patients who underwent surgical resection due to benign non-toxic goiter. They found a recurrence rate of 35% detected by ultrasonography during a median follow-up of 10 years (27). In a retrospective study, the assessment of 112 individuals 30 years after surgery showed rates of disease recurrence of 40–45% during a 30-year follow-up period (28). In another study, the recurrence rate of the goiters was 18% when assessed by ultrasonography 9 years after surgery (29).

This high probability of goiter recurrence after subtotal thyroidectomy resulted in a preventive use of LT4. However, only one (30) out of four randomized trials (27, 30–32) has demonstrated LT4 to be effective in this setting.

A cervical incision is often used to approach intrathoracic goiters; however, 10–30% of the patients require sternotomy or thoracotomy (33).

After total thyroidectomy, patients must start LT4 replacement at a dose of 1.4–2.2 μg/kg/day (34). Adjustments in LT4 dose must be based on the patient’s age (35), according to the usual recommendations for patients with hypothyroidism. However, in patients undergoing partial thyroidectomy, treatment with LT4 should be implemented after the establishment of hypothyroidism and not preventively against goiter recurrence, because this benefit has not been confirmed in randomized studies (36).

The benefits and risks of a surgical procedure must be carefully considered, since the incidence of NNG increases with age, affecting particularly elderly individuals who often have other comorbidities. As discussed below, rhTSH-stimulated radioiodine may be a treatment alternative for these patients.

## Therapy with Radioiodine

Therapy with radioiodine may be recommended in cases of NNG affecting patients who refuse or have contraindications for surgery. Over the past years, this type of treatment has increased in patients with nodular goiter and is associated with a substantial decrease in glandular volume, reaching 30–40% in the first year, and 50–60% in the fourth year. Obstructive symptoms improve in most individuals (37), with reports of a single dose administered orally restoring euthyroidism over a period of 2–4 months (2).

Radioiodine for the treatment of goiter was introduced approximately three decades ago. In an initial report, 25 individuals with a mean glandular volume of 73 cm^3^, who received 100 μCi of radioiodine per gram of thyroid tissue corrected to 100% uptake in 24 h, showed an approximate reduction in goiter volume of 41% after 1 year of follow-up (38). The larger the volume of the gland and the lower the radioiodine uptake (RAIU), the higher should be the radioiodine activity to be administered. Subsequent studies have unanimously corroborated this observation (39, 40). Patients with very large goiters (>100 cm^3^) have a smaller decrease in glandular volume (about 35%) even with administration of similar radioiodine doses (37).

In some European countries such as Denmark and the Netherlands (to some degree), radioiodine has currently replaced surgery as the treatment of choice for NNG (10). Depending on each country’s regulations, radioiodine administered for treatment purposes may be delivered in fractions on an outpatient basis, reducing the costs of hospitalization (41).

Some patients develop temporary mild thyrotoxicosis within the initial 2 weeks of treatment, and about 45% of them develop hypothyroidism, requiring thyroid hormone replacement for life (16). The occurrence of hyperthyroidism due to Graves’ disease associated with increased serum concentrations of TSH receptor antibodies has also been described in patients with increased baseline levels of thyroid peroxidase antibodies after radioiodine treatment for NNG (42).

Based on measurements of whole-body radiation exposure, the theoretical lifetime risk of development of cancer outside the thyroid gland has been calculated as 1.6%. This is due to the use of high doses of radioiodine in patients with very large goiters (the mean goiter volume used in the calculation was ~220 g). When administered to individuals aged 65 years or older, the estimated risk is ~0.5% (43).

### Recombinant Human TSH-Stimulated Radioiodine Therapy

Low isotope accumulation in inactive and partially suppressed areas around the nodule is a limitation of radioiodine treatment in patients with NNGs. This problem may be solved by increasing the RAIU in such nodular goiters (44). For this purpose, recent studies using rhTSH in preparation for radioiodine therapy have shown good results (45).

Several studies have assessed the adjuvant role of rhTSH in the radioiodine treatment of NNG. Administration of rhTSH is associated with a twofold to fourfold increase in RAIU by the thyroid (46). It should be noted that the baseline serum TSH may be a confounding factor since the increase in the 24-h thyroid RAIU correlates inversely with this variable. Since patients with NNG often present low serum TSH, the radioiodine is only taken up by some “hot” areas encircled by suppressed thyroid tissue that is inactive on scintigraphy. This phenomenon can be explained by a suppression of the paranodular parenchyma as a consequence of the low TSH level. Upon stimulation with rhTSH, these dormant areas, which concentrate radioiodine weakly, reactivate and eventually amplifies the effect of the radioiodine in the gland, promoting a further reduction in the volume of the goiter. In fact, rhTSH has been shown to distribute the radioiodine more homogeneously in the goiter, allowing a decrease in the dose of radioiodine to be administered (44). Most studies analyzing rhTSH in this setting have used fixed doses of radioiodine ranging from ~14 to ~42 mCi (37) and treatment with one or two rhTSH doses ranging from 0.1 to 0.3 mg administered 24 h prior to the radioiodine. However, the dose with ideal efficacy and safety is yet to be defined (47).

Combined therapy with rhTSH and radioiodine is well tolerated, and potential side effects that occur are similar to those observed in individuals receiving radioiodine alone. However, patients may experience a short increase in thyroid hormone levels within 48 h from the administration of radioiodine, leading to transient mild thyrotoxicosis, which may be followed by hypothyroidism during the first 30 days after the therapy (48, 49). Other acute adverse events that have been reported include painful transient thyroiditis, thyroid swelling, compression of the trachea, and, often, heart-related symptoms. Administration of glucocorticoids and β-blockers may help minimize these symptoms (50).

Recent studies (37) have shown that these adverse events may be dependent on the dose and are negligible with lower doses of rhTSH. Bonnema et al. (44) have shown that the ideal rhTSH dose to improve treatment with radioiodine may be about 0.03–0.1 mg. This dose range increases the thyroid RAIU substantially, with a minimal risk of thyroid swelling and transient thyrotoxicosis.

Studies assessing the long-term adverse effects of the combined use of rhTSH and radioiodine therapy showed an increased rate of permanent hypothyroidism. Three randomized controlled studies have reported 1-year rates of permanent hypothyroidism between 21 and 65% in rhTSH-pretreated patients compared with rates between 7 and 21% in patients in whom rhTSH was not administered (51–53). Due to its effect on the thyroid, rhTSH can potentially trigger an autoimmune response. Following treatment with radioiodine stimulated with rhTSH, 8 of 15 subjects with NNG developed antiperoxidase antibodies. However, no differences in autoimmunity were observed at 12 months between the subjects who underwent therapy with radioiodine alone versus those pretreated with rhTSH before radioiodine (54).

No studies have specifically assessed the risk of malignancy after NNG treatment with radioiodine with rhTSH prestimulation. However, by increasing the uptake of radioiodine by the thyroid, rhTSH results in less radioactivity delivered to the thyroid and to the rest of the body, which potentially reduces the theoretical risk of radioiodine-induced malignancy (37).

To avoid unintentional stimulation of the thyroid, a “modified-release rhTSH” (MRrhTSH) has been recently introduced. This compound has a slightly different serum profile than that of rhTSH, with a more delayed peak and, therefore, a possible lower risk of goiter enlargement. A phase II study of MRrhTSH use in patients with NNG undergoing radioiodine therapy was recently published. After 6 months, patients pre-stimulated with MRrhTSH 0.01 mg or placebo before radioiodine therapy had a 23% reduction in the goiter volume, whereas the reduction was 33% in individuals pre-stimulated with MRrhTSH 0.03 mg (55).

Both rhTSH and MRrhTSH are not approved by the US Food and Drug Administration or European Medicines Agency to treat NNG in association with radioiodine; so, their use for this purpose is currently off-label.

An alternative approach to increase the thyroid RAIU is to administer methimazole to stimulate the secretion of endogenous TSH. Albino et al. (56) pretreated nine patients with nodular goiter and subclinical hyperthyroidism with methimazole, aiming at increasing the serum level of TSH above 6 μU/mL. When this level was achieved, radioiodine 30 mCi was administered. The 24-h thyroid RAIU increased from 21 to 78%, and the mean goiter reduction at 1 year was 46%. In eight individuals (89%) with subclinical hyperthyroidism, the thyroid function normalized after 1 year. Five patients (56%) developed overt hypothyroidism, and no adverse clinical events were observed. However, whether a marginal hypothyroid state obtained with methimazole is as effective as rhTSH to increase the thyroid RAIU and to augment the reduction in goiter size after radioiodine therapy remains to be clarified with controlled trials.

In summary, individuals with NDG should receive clinical rather than surgical treatment. NNG is a highly prevalent disease, even in regions without iodine deficiency. Many individuals are asymptomatic, and when symptoms are present, the most common clinical manifestations result from local compressive effects. Individuals with NDG should be thoroughly evaluated to exclude malignancy. They should then receive individualized therapy after assessment of risks and benefits of each treatment option and discussion with their physicians. The first therapeutic option is total thyroidectomy, followed by treatment with radioiodine alone or after rhTSH stimulation to increase the radioiodine efficacy.

It is unclear if suppressive therapy with thyroid hormone is effective in patients with NNG. The choice of this therapy has decreased because of concerns regarding eventual long-term side events due to subclinical hyperthyroidism, and due to the fact after therapy interruption, most goiters grow again in size. All these therapies have potential advantages and disadvantages and are associated with short-term and long-term side events.

## Surgery or Radioiodine Therapy?

Table 3 presents various circumstances associated with the development of multinodular goiter, as well as the treatments (surgery or radioiodine) recommended in each case.

**Table 3:** **Circumstances of preferred thyroidectomy or radioiodine therapy in patients with multinodular goiter**.

	**Radioiodine**	**Surgery**
Small or moderate goiter size	++^a^	+
Prior thyroidectomy	++	+
Associated hyperthyroidism caused by nodular autonomy	++	+
Severe comorbidity	++	−
Suspicion of thyroid malignancy	−	++
Very large (>100 cm^3^) benign goiter without obstructive symptoms	+^b^	++
Severe tracheal compression	+	++
Need of rapid relief of goiter symptoms	+	++
Insufficient response to previous radioiodine therapy	+^c^	++
Retrosternal goiter or intrathoracic extension	++^d^	+

*++: first choice; +: second choice; −: no option*.

*^a^Observation may be considered in euthyroid patients with asymptomatic diffuse or benign nodular goiters*.

*^b^Treatment with rhTSH-stimulated radioiodine may be an option*.

*^c^A satisfactory result may be obtained with a second course of radioiodine*.

*^d^Retrosternal or intrathoracic goiters are often large, which makes surgery a preferable treatment*.

*Adapted with modifications from Ref. (44)*.

## Final Remarks

The therapeutic goals are to treat eventual thyroid dysfunction and reduce the size of the gland or prevent an additional increase in size. Without robust data from randomized studies, treatment decisions must be individualized according to the characteristics of the patients. Several individuals with large benign goiters may present symptoms of pressure, including dysphagia, neck tightness, or sensation of airway obstruction. Such patients frequently require surgical treatment to improve their symptoms. Asymptomatic patients in whom malignancy is excluded may be only observed. Controversy remains regarding the effectiveness of LT4 suppression implemented to reduce the size of the goiter. Radioactive iodine alone or with rhTSH is safe and effective and may be a reasonable therapeutic option.

Finally, which is the ideal treatment for benign NDG and NNG goiters? Once the diagnosis and indication for treatment of non-toxic goiter has been made, the treating physician and patient should discuss each of the treatment options, including the benefits, side effects, expected speed of recovery, drawbacks, and then decide on the best treatment modality for that particular patient, taking into account the patient’s age and comorbidities.

## Ethical Approval

This review did not include studies conducted by the author with the participation of humans or animals.

## Informed Consent

For this type of article, a formal consent is not required.

## Author Contributions

The author confirms being the sole contributor of this work and approved it for publication.

## Conflict of Interest Statement

The author declares that the research was conducted in the absence of any commercial or financial relationships that could be construed as a potential conflict of interest.
